# Effectiveness of Vortioxetine in Patients With Major Depressive Disorder in Real-World Clinical Practice: Results of the RELIEVE Study

**DOI:** 10.3389/fpsyt.2022.824831

**Published:** 2022-03-09

**Authors:** Gregory W. Mattingly, Hongye Ren, Michael Cronquist Christensen, Martin A. Katzman, Mircea Polosan, Kenneth Simonsen, Lene Hammer-Helmich

**Affiliations:** ^1^St Charles Psychiatric Associates & Midwest Research Group, St Charles, MO, United States; ^2^Medical Affairs, H. Lundbeck A/S, Valby, Denmark; ^3^START Clinic for Mood and Anxiety Disorders, Toronto, ON, Canada; ^4^Department of Psychology, Adler Graduate Professional School, Toronto, ON, Canada; ^5^Department of Psychiatry, Northern Ontario School of Medicine, Thunder Bay, ON, Canada; ^6^Department of Psychology, Lakehead University, Thunder Bay, ON, Canada; ^7^The Edward S. Rogers Sr. Department of Electrical and Computer Engineering, University of Toronto, Toronto, ON, Canada; ^8^Univ. Grenoble Alpes, Inserm, U1216, CHU de Grenoble Alpes, Grenoble Institut des Neurosciences (GIN), Grenoble, France

**Keywords:** major depressive disorder, vortioxetine, effectiveness, functioning, functional recovery, cognitive symptoms, real-world evidence, observational study

## Abstract

**Background:**

Randomized controlled clinical trials have shown vortioxetine to be efficacious and well tolerated for the treatment of major depressive disorder (MDD). The Real-Life Effectiveness of Vortioxetine in Depression (RELIEVE) study was undertaken to demonstrate the effectiveness and safety of vortioxetine for the treatment of MDD in routine clinical practice.

**Methods:**

RELIEVE was a 24-week, observational, prospective cohort study in outpatients with MDD initiating treatment with vortioxetine at their physician's discretion in routine care settings in Canada, France, Italy, and the USA (NCT03555136). The primary study outcome was patient functioning assessed by the Sheehan Disability Scale (SDS). Secondary outcomes included depression severity [9-item Patient Health Questionnaire (PHQ-9)], cognitive symptoms [5-item Perceived Deficits Questionnaire-Depression (PDQ-D-5)], and cognitive performance [Digit Symbol Substitution Test (DSST)]. Mixed models of repeated measures were used to assess change from baseline at week 24, adjusted for relevant confounders.

**Results:**

A total of 737 patients were eligible for inclusion in the full analysis set. Most patients (73.7%) reported at least one comorbid medical condition, 56.0% had comorbid anxiety and 24.4% had comorbid generalized anxiety disorder. Improvement in least-squares (LS) mean SDS score from baseline to week 24 was 8.7 points. LS mean PHQ-9, PDQ-D-5 and DSST scores improved by 7.4, 4.6, and 6.2 points, respectively. Adverse events were observed in 21.2% of patients [most commonly, nausea (8.2% of patients)].

**Conclusions:**

These results demonstrate the effectiveness and tolerability of vortioxetine for the treatment of MDD in a large and heterogeneous patient population representative of that encountered in routine clinical practice.

## Introduction

Depression is a debilitating psychiatric condition and a leading cause of disability, affecting more than 264 million people worldwide (1). Major depressive disorder (MDD) is associated with significant impairments in psychosocial functioning (2, 3). Functional impairment has been shown to persist even after resolution of other symptoms in patients with MDD (3), with residual functional impairment following remission of mood symptoms being predictive of subsequent relapse (4). Accordingly, functional recovery is increasingly recognized to be an important treatment goal in patients with MDD (5–8).

MDD is a multidimensional disorder that is associated with a wide range of emotional, physical, and cognitive symptoms. Data from the Sequenced Treatment Alternatives to Relieve Depression (STAR^*^D) study suggest that individual symptoms differ in terms of their impact on functioning in patients with MDD, with sad mood, concentration problems, fatigue, and loss of interest (anhedonia) found to have the greatest impact (9). Cognitive symptoms are common in patients with MDD, with deficits observed across multiple domains including executive function, memory, and attention (10–13). Numerous studies have shown a significant association between cognitive symptoms and functioning in MDD, which appears to be independent of the severity of mood symptoms (14–18).

Vortioxetine is a multimodal antidepressant with a unique mechanism of action (19), approved by the US Food and Drug Administration for the treatment of adults with MDD in 2013. Acting as an inhibitor of the serotonin transporter as well as a modulator of several serotonin receptor subtypes, vortioxetine both directly and indirectly influences multiple neurotransmitter systems relevant to the neurobiology of MDD (19, 20). Results of a recent independent meta-analysis of clinical trial data show vortioxetine to have broad efficacy against the core symptoms of MDD (i.e., depression, cognitive symptoms, and anxiety) (21). These results confirm earlier meta-analyses showing vortioxetine to have broad efficacy across the spectrum of symptoms experienced by patients with MDD (22–26), including anhedonia (27) and physical symptoms of depression (28). A dose–response relationship has been observed for improvements in both depressive symptoms and overall functioning, with greatest effects seen at a vortioxetine dosage of 20 mg/day (21, 29, 30).

Randomized controlled clinical trials are essential to demonstrate efficacy and tolerability of new antidepressants, but due to the regulatory requirements for clinical trial eligibility criteria, enrolled patients may not be fully representative of those likely to be encountered in routine clinical practice in terms of demographics, clinical characteristics, and comorbidities. Consequently, there is a need to confirm the results of regulatory studies in the more diverse patient population likely to be seen in clinical care settings, including those with multiple physical and psychiatric comorbidities.

The Real-Life Effectiveness of Vortioxetine in Depression (RELIEVE) study was undertaken to assess the effectiveness of vortioxetine in patients with MDD treated in routine clinical care settings. This study aimed to provide novel insights into the effectiveness of vortioxetine by focusing on the patient's own assessment of their functioning in daily life and the improvement experienced during treatment with vortioxetine using a validated and easy-to-use scale that is widely used for the assessment of overall functioning in patients with MDD. The primary study objective was to assess the effect of vortioxetine on self-reported patient functioning after 6 months of treatment. Secondary objectives included evaluation of treatment effects on depressive symptoms, cognitive symptoms and performance, sexual function, and health-related quality of life.

## Methods

### Study Design and Participants

RELIEVE was a 24-week, multinational, observational, prospective cohort study in outpatients with MDD initiating treatment with vortioxetine (NCT03555136). Eligible patients were aged ≥18 years, experiencing a major depressive episode (MDE) according to local diagnostic criteria, being treated by a general practitioner or at a psychiatric outpatient practice, and had been prescribed vortioxetine as treatment for the current MDE at their physician's discretion according to the local approved label. Patients with schizophrenia, bipolar disorder, substance abuse, or any neurodegenerative disease significantly impacting cognitive function were ineligible for study participation. Patients considered at significant risk of suicide or who had attempted suicide within the last 6 months were also excluded. As this was a real-world study, participating patients were permitted to receive other pharmacotherapy for MDD and/or other psychoactive medications during the study period. There were no exclusion criteria for concomitant medications.

Study assessments were performed at routine clinic visits at baseline and after 12 and 24 (±4) weeks of vortioxetine treatment. The study was conducted in accordance with the Declaration of Helsinki and Good Clinical Practice guidelines. Local ethics committee approval for the study was obtained at all participating sites and all patients provided written informed consent before study participation.

### Study Assessments

Functioning was assessed using the Sheehan Disability Scale (SDS). The SDS is a well-validated, short and simple tool that is widely used for the assessment of functioning in patients with MDD (31, 32). It is easily administered in clinical practice settings without disrupting the flow of routine care. This brief self-report measure assesses the degree of functional impairment experienced by patients over the previous 7 days in three key areas of their daily lives—namely, at work or school, in their family life and home responsibilities, and in their social life and leisure activities. The level of impairment for each domain is rated using a visual analog scale ranging from 0 (not at all) to 10 (very severe). Scores from the individual domains are combined to generate the SDS total score, ranging from 0 (unimpaired) to 30 (highly impaired). A reduction (i.e., improvement) in SDS total score of ≥4 points is considered meaningful for patients (32). Response was defined as SDS total score ≤ 12 points (32, 33). Work productivity measures (absenteeism and presenteeism) were derived from SDS scores based on days lost or underproductive for the working population. The number of days taken as sick leave was also assessed (during the past 12 weeks at baseline and since last visit at weeks 12 and 24).

Severity of depressive symptoms was assessed by patients using the 9-item Patient Health Questionnaire (PHQ-9) and by clinicians using the Clinical Global Impression (CGI) scale. The PHQ-9 assesses the severity of depressive symptoms experienced over the previous 2 weeks (34). PHQ-9 total score ranges from 0 to 27; scores of 5, 10, 15, and 20 points represent thresholds for mild, moderate, moderately severe, and severe depressive symptoms, respectively (34). The CGI–Severity (CGI-S) scale provides a measure of overall disease severity over the past 7 days; scores range from 1 (normal, not at all ill) to 7 (among the most extremely ill patients) (35, 36). The CGI–Improvement (CGI-I) scale assesses change in overall disease severity from baseline, with scores ranging from 1 (very much improved) to 7 (very much worse) (35, 36).

Severity of self-reported cognitive symptoms over the previous 7 days was assessed using the 5-item version of the Perceived Deficits Questionnaire-Depression (PDQ-D-5) (37, 38). The total PDQ-D-5 score ranges from 0 to 20, with higher scores indicative of more severe cognitive symptoms. Cognitive performance was assessed using the Digit Symbol Substitution Test (DSST) (39). This neuropsychological coding test involves the substitution of simple symbols for digits. The number of correct symbols substituted during a 90-s period is recorded; total score ranges from 0 to 133, with higher scores indicating better cognitive performance.

Sexual functioning was evaluated using the patient-reported Arizona Sexual Experience Scale (ASEX) (40). This 5-item scale assesses sex drive, arousal, vaginal lubrication/penile erection, ability to reach orgasm, and satisfaction from orgasm. The possible total score ranges from 5 to 30, with higher scores indicating greater sexual dysfunction.

Health-related quality of life was assessed using the EuroQoL 5 dimensions 5 levels questionnaire (EQ-5D-5L) (41). This widely used self-report instrument covers five health dimensions: mobility, self-care, usual activities, pain/discomfort, and anxiety/depression. There are five response levels: no problems, slight problems, moderate problems, severe problems, and unable to/extreme problems. A utility index score is derived from the responses, with a value of 1 indicating perfect health, 0 indicating a state equivalent to being dead, and <0 indicating a state considered worse than being dead. Patients also assessed their health state on a visual analog scale ranging from 0 (worst possible health) to 100 (best possible health).

Adverse events (AEs)/adverse drug reactions spontaneously reported by the patient or observed by the investigator were to be reported to the study sponsor or relevant authorities according to local regulations. AEs were summarized by lowest level Medical Dictionary for Regulatory Activities (Version 23.1) preferred terms.

### Statistical Analysis

The primary objective of this study was to quantify improvement from baseline in patient functioning assessed by SDS score. The number of enrolled patients was sufficient to assess the hypothesis of observing improvement in SDS score. Effectiveness assessments were conducted in all eligible patients who initiated treatment with vortioxetine ≤ 7 days before the study baseline visit and who had a valid baseline and at least one post-baseline assessment (full analysis set). The safety population comprised all patients who provided informed consent and initiated treatment with vortioxetine for MDD.

Data were collected during the study visits and/or from patient medical records; missing data were not imputed. Counts and percentages are presented for categorical variables and summary statistics for continuous variables. The primary study endpoint was mean change from baseline in SDS total score assessed at weeks 12 and 24. In patients who did not work or study during the follow-up period for reasons unrelated to MDD, an SDS work/school domain score was imputed for the calculation of SDS total score based on the average of the other two SDS domain scores, as previously described (33). This imputation was only made for the calculation of SDS total score; the work/school domain score was calculated without imputation. Secondary endpoints included mean change from baseline at weeks 12 and 24 for SDS domain scores and PHQ-9, CGI-S, CGI-I, PDQ-D-5, DSST, EQ-5D-5L, and ASEX scores.

For all endpoints (except CGI-I, as this score itself represents the change from baseline), mean change from baseline was assessed using a linear mixed model for repeated measures with an unstructured covariance matrix and the response variable and visit (baseline, week 12, and week 24) as the fixed effect. Analyses were performed both unadjusted with the above factors, as well as adjusted for clinically relevant covariates including age (continuous), sex, education level, duration of MDE at baseline (continuous), baseline comorbidities (mental disorder [yes/no] and physical disorder [yes/no]), and baseline depression severity (PHQ-9 score, continuous; except for the model for PHQ-9). Results of the adjusted analyses are reported as estimated least-squares (LS) means, with standard errors (SEs), 95% confidence intervals, and *p*-values. Analyses were performed for the overall study population, in the subgroup of patients with moderately severe to severe depression at baseline (PHQ-9 score ≥15), and by vortioxetine treatment line for the current MDE (first, second, and third or later line).

Safety variables were summarized over the entire study period; percentages were calculated using the safety population as denominator. All statistical analyses were performed using R version 3.6.1 (42). For all statistical tests, the significance level was set at 0.05.

## Results

### Study Population

This study was conducted between November 2017 and January 2021 at 103 sites in four countries (nine in Canada, 33 in France, 25 in Italy, and 36 in the USA). Of the 994 patients who provided informed consent for study participation, 985 met the criteria for inclusion in the safety population (three patients were excluded as they did not start treatment with vortioxetine and six due to missing data) and 737 met the criteria for inclusion in the full analysis set (three patients did not meet the study eligibility criteria, ten initiated vortioxetine >7 days before the baseline visit and 235 did not attend at least one post-baseline visit within the specified time period). The majority (84.8%) of patients included in the full analysis set completed the study. Of the 112 patients who did not complete the study, most (80.4%) were lost to follow-up or withdrew consent.

Baseline patient demographics are shown in Table 1. The mean age of the study population was 49.3 years and 115 patients (15.6%) were aged >65 years. The majority of patients were overweight or obese (61.7% of those with available body-mass index data) and most patients (73.7%) reported at least one comorbid medical condition [most commonly, sleep disorders (22.0%), cardiovascular disease (16.7%), chronic pain (8.5%) and diabetes (7.2%)]. Fifty-six percent of patients had comorbid anxiety and 24.4% had comorbid generalized anxiety disorder.

**Table 1:** Patient demographic characteristics at baseline (full analysis set).

**Characteristic**	**Full analysis set**
	**(*N* = 737)**
Age (years), mean ± SD	49.3 ± 15.4
>65 years, *n* (%)	115 (15.6)
Female, *n* (%)	473 (64.2)
Country, *n* (%)	
Canada	76 (10.3)
France	184 (25.0)
Italy	231 (31.3)
USA	246 (33.4)
Race/ethnicity	
White/Caucasian	597 (81.0)
Hispanic/Latino	13 (1.8)
Black/African American	21 (2.8)
Asian	6 (0.8)
Unspecified	100 (13.6)
BMI (kg/m^2^), mean ± SD	27.5 ± 6.0
Comorbidities, *n* (%)	
≥1 comorbidity	543 (73.7)
Overweight/obese (BMI ≥ 25.0 kg/m^2^)	448 (61.7)^a^
Anxiety	413 (56.0)
Sleep disorder	162 (22.0)
Cardiovascular disease	123 (16.7)
Time since onset of current anxiety (years), mean ± SD	9.6 ± 11.1
Living status, *n* (%)	
Living with others	560 (76.0)
Occupation, *n* (%)	
Full/part time work or school	411 (55.8)
Non-working	326 (44.2)

*BMI, body-mass index; SD, standard deviation*.

a *Percentage based on the number of patients with available data (n = 726)*.

Disease characteristics at baseline are shown in Table 2. Most patients (79.2%) reported at least one previous MDE (mean, 4.4 previous episodes). Mean duration of MDD was 11.2 years, and mean duration of the current MDE was 47 weeks. Baseline assessments showed patients to have impaired functioning across all SDS domains, moderate-to-severe depressive and cognitive symptoms, impaired sexual function, and impaired health-related quality of life.

**Table 2:** Patient clinical characteristics at baseline (full analysis set).

**Characteristic**	**Full analysis set (*N* = 737)**
Time since MDD diagnosis (years), mean ± SD	11.2 ± 12.1
Median (range)	7.0 (0–58)
Duration of current MDE (weeks), mean ± SD	47.0 ± 140.8
≤ 14 weeks, *n* (%)	409 (55.6)
>14 weeks, *n* %	326 (44.4)
No. of previous MDEs, mean ± SD	4.4 ± 9.1
0 previous MDEs, *n* (%)	151 (20.5)
1 previous MDE, *n* (%)	157 (21.4)
2+ previous MDEs, *n* (%)	427 (58.1)
Vortioxetine treatment line for current MDE, *n* (%)	
1st line	321 (43.6)
2nd line	257 (34.9)
≥3rd line	159 (21.6)
Vortioxetine dose (mg/day), mean ± SD	
Baseline	9.6 ± 5.5
Week 24	12.9 ± 6.5
Clinical assessment scores, mean ± SD	
SDS total score	19.6 ± 6.6
Work/school domain	6.3 ± 2.7
Family life/home responsibilities domain	6.6 ± 2.4
Social life/leisure activities domain	6.7 ± 2.4
PHQ-9	16.5 ± 5.5
PDQ-D-5	11.2 ± 4.9
DSST	42.6 ± 16.8
EQ-5D-5L utility score	0.65 ± 0.16
ASEX	22.1 ± 6.5
CGI-S	4.3 ± 0.9
Work productivity measures, mean ± SD	
Absenteeism (days/week)	2.2 ± 2.7
Presenteeism (days/week)	4.0 ± 2.6
Sick leave (days in the past 12 weeks)	26.6 ± 29.1

*Absenteeism, SDS days lost for working population; ASEX, Arizona Sexual Experience Scale (score range 5–30); CGI-S, Clinical Global Impression–Severity (score range 1–7); DSST, Digit Symbol Substitution Test (score range 0–133); EQ-5D-5L, EuroQoL 5 dimensions 5 levels utility index (score range 0–1); MDD, major depressive disorder; MDE, major depressive episode; PDQ-D-5, 5-item Perceived Deficits Questionnaire-Depression (score range 0–20); PHQ-9, 9-item Patient Health Questionnaire (score range, 0–27); Presenteeism, SDS days underproductive for working population; SD, standard deviation; SDS, Sheehan Disability Scale*.

Vortioxetine was initiated as first-line treatment for the current MDE in 43.6% of patients. Of the 416 patients who had received treatment for the current MDE before the study baseline visit, 80.0% were switching to vortioxetine due to lack of effectiveness of prior antidepressant therapy and 10.8% due to lack of tolerability. Data on previous antidepressant treatment for the current MDE prior to initiation of vortioxetine were available for 266 patients. The most common previous antidepressants reported to have been received for the current MDE were escitalopram [71 patients (26.7% of those with available data)], sertraline [61 patients (22.9%)], bupropion [44 patients (16.5%)], paroxetine [43 patients (16.2%)], venlafaxine [35 patients (13.2%)], and duloxetine [35 patients (13.2%)]. Patients may have reported more than one previous antidepressant.

A total of 170 patients (23.1%) were receiving another antidepressant in addition to vortioxetine at baseline, most commonly, bupropion (received by 6.4% of all patients). A total of 410 patients (55.6%) were receiving at least one other psychotropic medication at baseline. The most common types of concomitant psychotropic medications were anxiolytics (received by 37.4% of all patients), hypnotics (14.8%), and antipsychotics (10.3%).

Data on the starting dose of vortioxetine were available for 712 patients. The starting dose of vortioxetine was 10 mg/day in 384 patients (53.9% of those with available data). The starting dose of vortioxetine was 5 mg/day in 225 patients (31.6%), 15 mg/day in 39 patients (5.5%), and 20 mg/day in 64 patients (9.0%). Mean (SD) vortioxetine dose was 9.6 (5.5) mg/day at baseline and 12.9 (6.5) mg/day at week 24. Fifty patients (6.8%) had discontinued treatment with vortioxetine by week 24.

### Effectiveness

**Figure 1:**
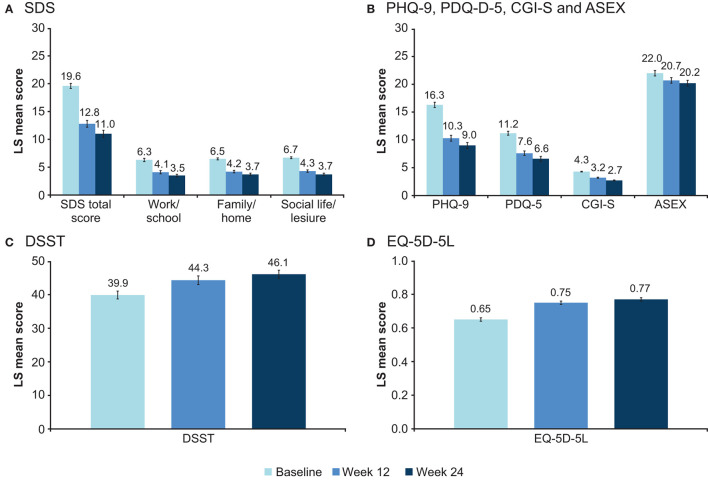
Adjusted LS mean (95% CI) score at baseline and after 12 and 24 weeks for **(A)** SDS total and domain scores, **(B)** PHQ-9, PDQ-D-5, CGI-S, and ASEX, **(C)** DSST, and **(D)** EQ-5D-5L scores (full analysis set). ASEX, Arizona Sexual Experience Scale (score range 5–30); CGI-S, Clinical Global Impression–Severity (score range 1–7); CI, confidence interval; DSST, Digit Symbol Substitution Test (score range 0–133); EQ-5D-5L, EuroQoL 5 dimensions 5 levels utility index (score range 0–1); LS, least-squares; PDQ-D-5, 5-item Perceived Deficits Questionnaire-Depression (score range 0–20); PHQ-9, 9-item Patient Health Questionnaire (score range 0–27); SDS, Sheehan Disability Scale.

LS mean scores for all effectiveness outcomes at baseline, week 12, and week 24 are shown in Figure 1. LS mean changes from baseline after 24 weeks of vortioxetine treatment for all scores are shown in Table 3.

**Table 3:** Adjusted LS mean change (SE) from baseline to week 24 for primary and secondary study endpoints (full analysis set).

**Variable**	**Adjusted LS mean change (SE) from baseline to week 24**	
	Full cohort^*^	Patients with moderately severe to severe depression at baseline (PHQ-9 score ≥15)^*^
SDS total score	−8.7 (0.3)	−9.9 (0.4)
Work/school	−2.9 (0.2)	−3.3 (0.2)
Family life/home responsibilities	−2.9 (0.1)	−3.3 (0.2)
Social life/leisure activities	−3.0 (0.1)	−3.3 (0.2)
PHQ-9	−7.4 (0.3)	−9.4 (0.3)
CGI-S	−1.5 (0.1)	−1.7 (0.1)
PDQ-D-5	−4.6 (0.2)	−5.5 (0.3)
DSST	6.2 (0.5)	5.8 (0.7)
ASEX	−1.8 (0.2)	−2.1 (0.3)
EQ-5D-EL	0.13 (0.01)	0.15 (0.01)

*ASEX, Arizona Sexual Experience Scale (score range 5–30); CGI-S, Clinical Global Impression–Severity (score range 1–7); DSST, Digit Symbol Substitution Test (score range 0–133); EQ-5D-5L, EuroQoL 5 dimensions 5 levels utility index (score range 0–1); LS, least-squares; MDD, major depressive disorder; MDE, major depressive episode; PDQ-D-5, 5-item Perceived Deficits Questionnaire-Depression (score range 0–20); PHQ-9, Patient Health Questionnaire (score range, 0–27); SE, standard error*.

* *All changes, p <0.0001 vs. baseline*.

**Figure 2:**
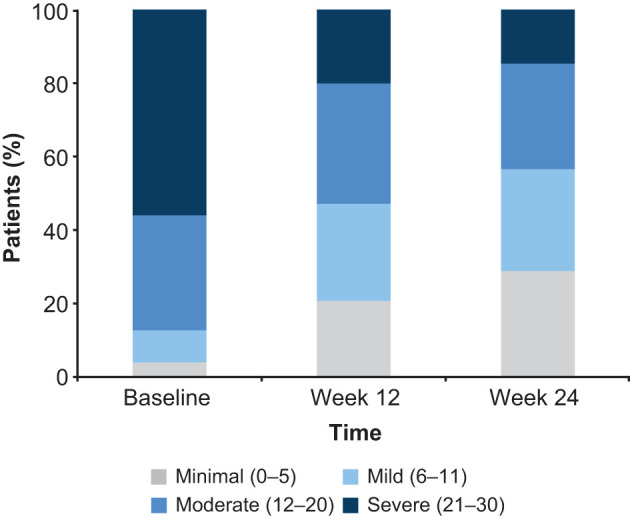
Proportion of patients according to severity of functional impairment (i.e., Sheehan Disability Scale total score) at baseline and after 12 and 24 weeks of vortioxetine treatment (full analysis set).

Clinically meaningful and sustained improvement in patient functioning was seen over the 24 weeks of vortioxetine treatment. LS mean for the SDS total score decreased (i.e., improved) from 19.6 points at baseline to 12.8 points at week 12 and 11.0 points at week 24 (Figure 1). This corresponds to a change in LS mean (SE) SDS total score from baseline of −6.9 (0.3) points at week 12 and of −8.7 (0.3) points after 24 weeks of vortioxetine treatment (both *p* < 0.0001). Significant reductions in all SDS domain scores were observed at both timepoints (*p* < 0.0001 for all changes at weeks 12 and 24 vs baseline). The proportion of patients experiencing a meaningful improvement in SDS total score (i.e., improvement ≥4 points) was 62.7% at week 12 and 71.8% at week 24. The proportion of patients with severe functional impairment (SDS total score >20) decreased from 51.5% at baseline to 20.1% at week 12 and 14.7% at week 24 (Figure 2). After 24 weeks of vortioxetine treatment, 56.6% of patients reported mild or minimal functional impairment compared with only 11.7% of patients at study baseline. The proportion of patients with SDS total score ≤ 12 (i.e., SDS responders) increased from 15.0% at baseline to 51.8% at week 12 and 60.5% at week 24.

**Figure 3:**
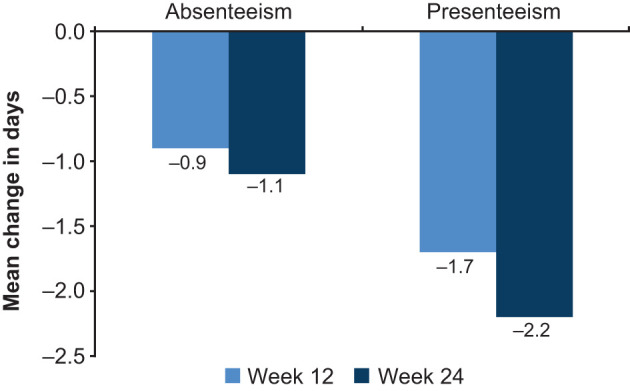
Change from baseline in days/week for SDS measures of work productivity at weeks 12 and 24 (full analysis set). Absenteeism, SDS days lost for working population; presenteeism, SDS days underproductive for working population; SDS, Sheehan Disability Scale.

Improvements from baseline were seen at week 24 for all work productivity measures (sick leave, absenteeism, and presenteeism). At week 24, the mean reduction in sick leave from baseline during the preceding 12 weeks was 1.7 days. Mean reductions in absenteeism (work days lost) and presenteeism (work days underproductive) after 24 weeks of vortioxetine treatment were 1.1 and 2.2 days/week, respectively (Figure 3).

Statistically significant and sustained improvements in patient- and clinician-rated measures of depression severity, cognitive symptoms and cognitive performance, sexual function, and health-related quality of life were also seen over the 24 weeks of vortioxetine treatment (Figure 1). For all assessments, LS mean changes from baseline were statistically significant at both week 12 and week 24 (all *p* < 0.0001) (Table 3). The proportion of patients with severe disease (CGI-S score 5–7) decreased from 41.0% at baseline to 10.7% at week 12 and 6.1% at week 24. The proportion of patients with mild disease (CGI-S score 1–3) was 73.6% after 24 weeks of vortioxetine treatment compared with 15.1% at baseline. Mean (SD) CGI-I score was 2.7 (1.0) points at week 12 and 2.5 (1.1) points at week 24, both corresponding to “much improved” vs. baseline. The proportion of patients with a CGI-I score indicative of improvement (i.e., CGI-I score of 1, 2, or 3) was 81.8% at week 12 and 83.5% at week 24.

The proportion of patients meeting the definition for sexual dysfunction (i.e., ASEX total score ≥19, ASEX score ≥5 on any item, or ASEX score ≥4 on any three items) decreased from 77.8% at baseline to 68.1% after 12 weeks of vortioxetine treatment and 63.4% after 24 weeks. Of the 107 patients who did not meet the definition for sexual dysfunction at baseline, 25.2% had sexual dysfunction at week 24.

Significant improvement in health-related quality of life was also observed over the 24 weeks of vortioxetine treatment (*p* < 0.0001). At 24 weeks, improvement in LS mean (SE) EQ-5D utility score from baseline was 0.13 (0.01) points. Mean (SD) EQ-5D VAS score increased from 50.6 (20.8) points at baseline to 64.3 (20.9) after 12 weeks and 68.9 (19.5) after 24 weeks of vortioxetine treatment.

### Effect of Baseline Depression Severity

In the subgroup of patients with moderately severe to severe depression at baseline (i.e., PHQ-9 score ≥15), statistically significant and sustained improvements in functioning and all secondary endpoints were seen over the 24 weeks of vortioxetine treatment (Table 3). For all endpoints except DSST score, the mean change from baseline was numerically greater in the subgroup of patients with moderately severe to severe depression at baseline than in the overall study population.

### Effect of Vortioxetine Treatment Line

**Figure 4:**
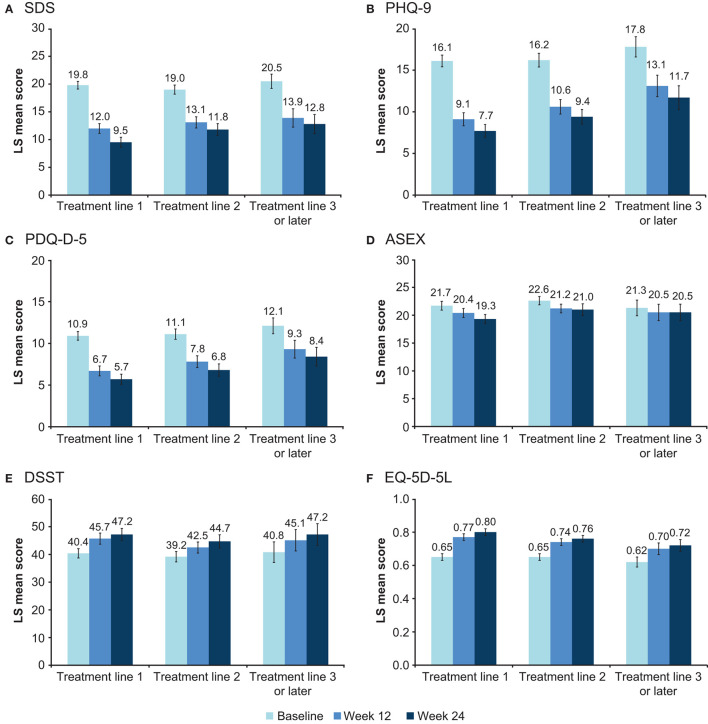
Adjusted LS mean (95% CI) score at baseline and after 12 and 24 weeks according to vortioxetine treatment line for **(A)** SDS total score, **(B)** PHQ-9, **(C)** PDQ-D-5, **(D)** ASEX, **(E)** DSST, and **(F)** EQ-5D-5L scores (full analysis set). ASEX, Arizona Sexual Experience Scale (score range 5–30); CI, confidence interval; DSST, Digit Symbol Substitution Test (score range 0–133); EQ-5D-5L, EuroQoL 5 dimensions 5 levels utility index (score range 0–1); LS, least-squares; PDQ-D-5, 5-item Perceived Deficits Questionnaire-Depression (score range 0–20); PHQ-9, 9-item Patient Health Questionnaire (score range, 0–27); SDS, Sheehan Disability Scale.

Sustained improvement in functioning and all secondary endpoints was seen over the 24 weeks of treatment, irrespective of vortioxetine treatment line (Figure 4). For all effectiveness endpoints, change from baseline was generally numerically greater in patients receiving vortioxetine as first-line treatment for the current MDE compared with subsequent treatment lines. At week 24, change in LS mean (SE) SDS total score from baseline was −10.3 (0.5) points in patients receiving vortioxetine as first-line treatment, −7.2 (0.6) points in those receiving vortioxetine as second-line treatment, and −7.7 (0.7) points in patients for whom this was third- or later-line treatment (all *p* < 0.0001). At week 24, LS mean changes from baseline were statistically significant for all other effectiveness endpoints across all vortioxetine treatment lines (all *p* < 0.0001), with the exception of ASEX score in patients receiving vortioxetine as third- or later-line treatment (not significant).

### Safety

In total, 21.2% of patients reported at least one AE over the 24 weeks of vortioxetine treatment. The most commonly reported AEs were nausea (81 patients; 8.2%), headache (15 patients; 1.5%), pruritus (15 patients; 1.5%), and anxiety (14 patients; 1.4%). No other AEs were reported by >1% of patients. In terms of AEs that have been associated with other antidepressants, insomnia was reported by five patients (0.5%), weight increase by four patients (0.4%), decreased libido by three patients (0.3%), loss of libido by one patient (0.1%), and sexual dysfunction by one patient (0.1%). Of the 29 serious AEs reported in 23 patients, three were considered to be at least possibly related to treatment (pancreatitis, serotonin syndrome, and suicidal ideation, each reported in a single patient). Two patients died during the study (one due to pneumonia and one due to chronic obstructive pulmonary disease); neither death was considered to be related to study treatment.

## Discussion

Functional recovery is recognized to be an important treatment goal in patients with MDD (5–8); however, the potential of antidepressant therapies to directly improve functional outcomes has not been extensively evaluated in routine care settings to date. In this real-world study in patients with MDD treated with vortioxetine in routine practice settings, significant and sustained improvements were seen in overall functioning and across all SDS domains over a period of 6 months. Meaningful improvement in SDS total score was experienced by almost two-thirds of all patients after 12 weeks of vortioxetine treatment and by approximately three-quarters after 24 weeks. Patients also reported missing significantly fewer days of work after 24 weeks of vortioxetine treatment and, perhaps even more importantly, patients were more productive while at work. Significant and sustained improvements in depression severity, cognitive symptoms, cognitive performance, and health-related quality of life were also observed over the 24 weeks of vortioxetine treatment, both in the overall patient population and in the subgroup of patients with moderately severe to severe depressive symptoms at baseline (PHQ-9 score ≥15 points). Clinically meaningful improvement was seen across functioning and all other effectiveness outcomes in patients receiving vortioxetine as first-line therapy for the current MDE and in those who had received one or more prior antidepressants for the current MDE, with greatest improvement seen when vortioxetine was used as a first-line treatment.

The level of adherence to treatment with vortioxetine achieved in conditions of routine clinical practice in this study is encouraging. The majority of patients (85%) included in the full analysis set completed the study, and most of those who did not complete the study (80%) were lost to follow-up or withdrew consent. Long-term treatment with vortioxetine was well tolerated. Consistent with the established tolerability profile of vortioxetine, nausea was the most commonly reported AE (19). The overall incidence of AEs in this study was lower than seen in pivotal randomized controlled clinical trials (43). No new safety issues were reported and the incidence of AEs that have been associated with other antidepressants, such as sexual dysfunction, weight gain, and insomnia (44–47), was low (<0.5%). Sexual side effects are one of the main reasons for poor adherence and treatment discontinuation in patients receiving selective serotonin reuptake inhibitors (SSRIs) (44, 45). In the present study, minimal changes in ASEX score were observed over the 6 months of vortioxetine treatment. Treatment-emergent sexual dysfunction was observed in 25% of patients who were unaffected at baseline. In contrast, sexual dysfunction has been reported in up to 50% of untreated patients with MDD (48), and up to 80% of those treated with SSRIs (49).

The results of this large international study are in keeping with those of other small-scale studies in patients with MDD treated with vortioxetine in routine care settings (16, 50–53). Similarly, a meta-analysis of data from nine short-term randomized placebo-controlled clinical trials of vortioxetine 5–20 mg found overall functioning to be significantly improved in patients with MDD treated with vortioxetine, with greatest effects seen at a dosage of 20 mg/day (50). The observed improvement in cognitive symptoms during treatment with vortioxetine in the present study is also consistent with previous findings both in open-label studies (16, 50–53) and randomized controlled clinical trials (24, 54–58). It seems reasonable to assume that the observed improvement in functioning seen in patients with MDD during treatment with vortioxetine may at least in part be due to the beneficial effect of treatment on cognitive symptoms. Indeed, previous studies have shown an independent association between severity of cognitive symptoms and impairment in functioning and workplace performance in patients with MDD (14–18). A significant association between the severity of patient-reported cognitive symptoms (assessed using the PDQ-D-5) and health-related quality of life (assessed using the EQ-5D) has also been previously reported (59).

In the present study, just over half of all patients had comorbid anxiety and almost one-quarter had a concomitant diagnosis of generalized anxiety disorder. Concurrent anxiety symptoms are common in patients with MDD (60–65) and have been shown to contribute to poor response to antidepressant treatment, lower rates of remission, increased risk of recurrence, and greater functional impairment (60, 61, 63, 64, 66–68). Significant correlation between severity of anxiety symptoms and functional outcomes has also been reported in patients with MDD (69, 70). Vortioxetine has been shown to be effective for the treatment of MDD in patients with high levels of anxiety symptoms (16, 23), and in patients with comorbid anxiety disorders (71–73). This beneficial effect of vortioxetine on anxiety symptoms may at least in part have contributed to the improvement in functioning and health-related quality of life observed in the present study.

The main strength of this study is that it was conducted in daily medical practice in a large and heterogeneous patient population, thereby generating valuable evidence to complement that derived from randomized controlled trials. Many patients had comorbid medical conditions and anxiety disorders, and the study population comprised both patients receiving vortioxetine as a first-line treatment for the current MDE and those switching from other antidepressants. All necessary concomitant medications were allowed, and participating patients were also permitted to receive other pharmacotherapy for MDD and/or other psychoactive medications. As such, study findings can be considered generalizable to patients with MDD treated in routine care settings. In addition, patients in this study were followed for 6 months, reflecting the general need for patients with MDD to receive long-term treatment.

A further study strength is that functional impairment, MDD symptoms, and the effectiveness of vortioxetine were assessed using patient-reported outcome measures. This is in keeping with the increased awareness of the importance of addressing patient perspectives when managing mental health disorders such as MDD (74, 75). Clinician-rated scales, such as the Montgomery–Åsberg Depression Rating Scale or the Hamilton Depression Rating Scale, are often used to assess the efficacy of antidepressant treatment in clinical trial settings, while the Functioning Assessment Short Test (FAST) may be used for the clinical evaluation of functional impairment in patients with MDD. However, clinician-rated scales may not fully capture a patient's subjective experience of MDD and antidepressant treatment, and patients' perceptions of symptoms and treatment outcomes in MDD have been shown to differ from those of their clinicians (76–80). Furthermore, most clinician-rated scales that are short, easy and applicable for routine care are interview-based. As such, results of these assessments are also based on patient reports, albeit interpreted by the clinician. More objective functional tests would not be considered routine care in most countries.

Study limitations include the naturalistic and observational study design, potential selection bias (i.e., patients who do not feel that their condition is being adequately treated may be more likely to enroll in this type of study), and the lack of a placebo or active comparator. In response to the COVID pandemic, a critical management plan was implemented that allowed patients to have remote follow-up visits and complete some patient-reported outcome assessments at home if needed. However, this is not expected to have had a significant impact on the study results as only 22 patients did not complete their visits or completed their visits remotely.

## Conclusions

In summary, results of this large real-world study demonstrate the effectiveness and tolerability of vortioxetine for the treatment of MDD in a large and heterogeneous patient population representative of that encountered in everyday clinical practice. Patients with MDD treated with vortioxetine experienced clinically relevant improvements in functioning, depressive symptoms, cognitive symptoms and performance, and health-related quality of life over the 6-month treatment period, with greatest effects seen when vortioxetine was used as a first-line treatment.

## Data Availability Statement

The data supporting the findings of this study are included in the article, further inquiries can be directed to the corresponding author.

## Ethics Statement

This study involving human participants was reviewed and approved by local Ethics Committees at all participating sites. The patients/participants provided their written informed consent to participate in this study.

## Author Contributions

MCC was responsible for conceptualization and design of the study. LH-H developed the study protocol and responsible for the operationalization of the study. HR managed the study follow-up. GWM, MAK, and MP were involved in the acquisition of data. KS was responsible for data analysis. The authors are entirely responsible for the scientific content of this paper. All authors contributed to drafting, revising the article, data interpretation, and approved the submitted version.

## Funding

The RELIEVE study was funded by H. Lundbeck A/S, whose personnel contributed to the data analysis, review of the data, and review of the manuscript. Medical writing assistance was provided by Jennifer Coward of Piper Medical Communications, funded by H. Lundbeck A/S.

## Conflict of Interest

This study was funded by H. Lundbeck A/S, whose personnel contributed to the data analysis, review of the data, and review of the manuscript. GWM has served as researcher, speaker or consultant for AbbVie, Acadia, Akili, Alkermes, Axsome, Boehringer, Eisai, Emalex, Idorsia, Intracellular, Ironshore, Janssen, Lundbeck, Medgenics, Neos, NLS1 Pharma, Otsuka, Purdue, Reckitt Benckiser, Rhodes, Roche, Sage, Shire, Sunovion, Supernus, Takeda, Teva, and Tris. MAK has been a researcher, speaker or consultant for Allergan, Astellas, AstraZeneca, Bedrocan, Boehringer Ingelheim, Bristol-Myers Squibb, Eli Lilly, Eisai, Forest, Genuine Health, GlaxoSmithKline, Hoffman-La Roche, Janssen, Lundbeck, Organon, Perdue, Janssen, Janssen-Ortho, Merck, Organon, Otsuka, Pfizer, Purdue, Shire, Solvay, Sunovion, Takeda, and Tilray. MP has received honoraria for lecturing from Janssen, LivaNova, Lundbeck, Medtronic, and the Movement Disorder Society, and research support from Boston Scientific. MCC and LH-H are employees of H. Lundbeck A/S. HR and KS were employees of H. Lundbeck A/S at the time this study was conducted.

## Publisher's Note

All claims expressed in this article are solely those of the authors and do not necessarily represent those of their affiliated organizations, or those of the publisher, the editors and the reviewers. Any product that may be evaluated in this article, or claim that may be made by its manufacturer, is not guaranteed or endorsed by the publisher.
